# Perinatal outcome of twin pregnancies among mothers who gave birth in Adama Hospital Medical College, Central Ethiopia

**DOI:** 10.1371/journal.pone.0275307

**Published:** 2022-09-29

**Authors:** Mekonen Beyene, Meyrema Abdo Komicha, Hassen Hussien, Anwar Abdulwahed, Tahir Ahmed Hassen, Kedir Teji Roba

**Affiliations:** 1 Department of Public Health, Adama Hospital Medical College, Adama, Ethiopia; 2 Department of Nursing, College of Health Science, Arsi University, Assela, Ethiopia; 3 Department of Nursing, College of Health Science, Haramaya University, Dire Dawa, Ethiopia; Reggio Emilia Local Agency - IRCCS Advanced Technologies and Care Models in Oncology: Azienda Unita Sanitaria Locale - IRCCS Tecnologie Avanzate e Modelli Assistenziali in Oncologia di Reggio Emilia, ITALY

## Abstract

**Introduction:**

Twin pregnancy carries a high risk of pregnancy-related complications including adverse perinatal outcomes. Although evidence from international studies indicated an increased risk of adverse perinatal outcomes in twin pregnancies, little is known about the adverse perinatal outcomes in twin pregnancies and associated factors in Ethiopia. The purpose of this study was, therefore, to estimate the incidence of twin pregnancies and related-adverse perinatal outcomes and identify factors associated with adverse perinatal outcomes in twin pregnancies in Ethiopia.

**Methods:**

A hospital-based retrospective cross-sectional study was conducted among 322 mothers who gave twin birth at Adama Hospital Medical College between 08 July 2015 and 07 June 2017. In this study, the adverse perinatal outcome was defined as the presence of any of the following main conditions: low birth weight, preterm birth, stillbirth, low Apgar Scores, mal-presentation, Admission to neonatal Intensive Care Unit (NICU), and early neonatal deaths. The data were analyzed using SPSS version 20.0. Multivariable logistic regression was conducted to identify factors associated with adverse perinatal outcome at 95% CI or P-value of less than 0.05.

**Result:**

Of 10,850 births recorded in the hospital, 354 births were twins and 322 of these paired records had complete perinatal information. One hundred ninety-nine (61.8%) of the 322 paired birth records had at least one adverse perinatal outcome on one or both twins. Low birth weight was the most common perinatal outcome followed by preterm birth. After adjusting for confounding factors, younger maternal age (AOR = 4.1, 95% CI; 1.3, 12.5) and not having ultrasound scan during antenatal care (AOR = 2.0, 95% CI: 1.2, 3.1) were significantly associated with adverse perinatal outcomes.

**Conclusion:**

The incidence of adverse perinatal outcome in twin pregnancies was high, that is, in 61.8% of twin births, there was at least one adverse perinatal outcome on one or both twins. Moreover, younger maternal age at birth and not having an ultrasound scan during antenatal care were found to be strong predictors for the observed high incidence of adverse perinatal outcomes.

## Introduction

Over the last four decades, globally, the rate of twin pregnancy has been increased by a third (from 9.1 per 1000 births in 1980 to 12.0 per 1000 births in 2021), constituting one in 42 births or 1.6 million twin births each year [1]. The increment has mainly been attributed to the advancement of assisted reproductive technology [2–4] and postponement of parenthood to the later age [5–7], particularly in high countries. In Africa, although the practice of assisted reproductive technology and delaying childbearing age is not common, naturally conceived twin pregnancy is becoming more prevalent [8]. A current estimate suggested that about 80% of twin pregnancies occurred either in Africa or Asia [1].

Twin pregnancy results from a complex interaction of different factors such as maternal age, parity, family history of multiple pregnancies, and others [9, 10]. Due to inherent biological factors, a twin pregnancy is associated with various adverse perinatal outcomes and maternal obstetrics complications [11]. Preterm birth, low birth weight, twin to twin transfusion syndrome, perinatal deaths, and admission to neonatal intensive care unit (NICU) are among many adverse perinatal outcomes associated with twin pregnancy [12, 13]. For example, the rate of perinatal mortality among twin pregnancies could be up to six times higher compared to singleton pregnancies [11].

Maternal obstetrics complications such as hemorrhage, preeclampsia, and maternal deaths are also common among twin pregnancies [14, 15]. Further, twin pregnancies increase both medical and surgical interventions including caesarean section [16, 17]. Mothers with twin pregnancies are also about six times more likely to be hospitalized due to complications during pregnancy compared to those with singleton pregnancies, posing additional healthcare costs [18].

Despite this, in most developing countries including Ethiopia, little is known about the current estimate of twin pregnancy and related adverse perinatal outcomes. Thus, for the proper resource allocation and management of mothers with twin pregnancies, there is a need to estimate the incidence of twin pregnancies and related adverse perinatal outcomes and understand the associated factors. The purpose of this study, therefore, to estimate the incidence of adverse perinatal outcomes and identify factors associated among twin pregnancies in Ethiopia.

## Methods and materials

### Study design and setting

A hospital-based retrospective cross-sectional study was conducted among mothers who gave birth to twins at Adama Hospital Medical College (AHMC) between 08 July 2015 and 07 July 2017. Adama Hospital Medical College is one of the largest referral public hospitals located in the Eastern Shoa zone of Oromia Regional State in Ethiopia. The hospital provides both outpatient and inpatient services covering a five million population in the catchment area. The hospital provides obstetrics and gynecology services including antenatal care (ANC), labor and delivery services, and post-natal care. About 5000 mothers attended ANC services and 10, 850 births were registered during the period of 08 July 2015─ 07 June 2017.

#### Study population

The study population was all twin births recorded at Adama Hospital Medical College during the study period (08 July 2015 and 07 June 2017) and fulfilled the inclusion criteria.

#### Inclusion and exclusion criteria

Twin births were included if the birth occurred after 28 weeks of gestational age including a retained second twin. Gestational age at birth was determined using both the Last Normal Menstrual Period and Ultrasound. Birth records with incomplete information about labor and births were excluded from the study.

### Sample size determination and sampling method

The sample size was calculated using a single population proportion formula considering the following parameters: 95% confidence level that falls within a 4% margin of error, and 16% of mothers who had twin pregnancy. We reduced the margin of error to 4% to increase the quality of data as the reasonable estimate of the key proportion to be studied was less than 50%. Considering the aforementioned parameters the sample was found to be 322. Using the list of twin-births from medical records as a sampling frame, the twin-birth records of 322 were sampled by a simple random sampling method.

### Dependent variables

The dependent variable was the adverse perinatal outcome. In this study, the adverse perinatal outcome was defined as the presence of any of the following main conditions: low birth weight, preterm birth, stillbirth, low Apgar Scores, mal-presentation, Admission to neonatal Intensive Care Unit (NICU), and early neonatal deaths.

### Independent variables

The independent variables include demographic factors such as maternal place of residence, maternal age at birth, and obstetrics factors including parity, gravida, ANC follow-up, Ultrasound scan during pregnancy and labour, and gestational age at birth.

### Data collection procedure

Maternal and birth-related data were collected using various medical records such as birth/delivery logbooks, operation registration books, mother and newborn’s cards, and newborn admission and discharge registration books for those newborns admitted to NICU. All these medical records were reviewed and a structured checklist was used for the data extraction by the trained and authorized data collector.

#### Data quality assurance

Intensive training was given for the data collectors on the checklist and methods of data extraction. The data collection process, completeness, and consistency of every checklist were regularly monitored and checked by the principal investigator. The checklist used for the data extraction was pretested on 5% of the study sample at Assella Hospital adhering to the similar ethical guidelines that were followed in the actual study.

#### Data processing and analysis

The data were entered and analyzed using SPSS version 20.0. Descriptive statistics were used to describe the main and selected variables included in the study. Bivariate analyses were conducted to investigate the association between each independent factor and outcome variable. The factors which were statistically significant at a P-value of less than 0.05 in bivariate analyses were carried forward into multivariable logistic regressions, where they were adjusted for potential confounders. Finally, after controlling for potential confounders, variables that showed a significant association with the outcome variable at P-value of less than 0.05 were considered as independent predictors of adverse perinatal outcome.

### Ethical considerations

Prior to data collection, the study protocol was evaluated and approved by the Ethical Review Committee of Adama Hospital Medical College and the authors were given the approval letter. The Approval letter was then submitted to the department of obstetrics and gynaecology of the hospital to get access to the medical records. When reviewing medical records, confidentiality was strictly maintained by excluding any personal identifiers and by restricting data access only to the authorized people.

## Results

### Socio-demographic and obstetrics characteristics

A total of 10,850 births were recorded during the study period at Adama Hospital Medical College. Of these, twin-births accounted for 354 (3.26%). The mean maternal age at birth was 26.6 years (SD± 4.7years). About 44% of twin births were recorded within the age range of 25–29 years. Regarding obstetrics characteristics, the mean parity was 2.6 (SD± 1.7) and the majority of mothers (98.1%) had 2–4 para. Three hundred sixteen mothers had ANC follow up and ultrasound examinations were performed for 54% and 97.2% of mothers during ANC follow up and during labour, respectively (See Table 1 for the details).

**Table 1:** Socio-demographic factors & maternal health service utilization characteristics among mothers who gave twin birth at AHMC, Ethiopia.

Variables	Frequency	Percentage
**Place of Residence**		
Adama town	130	40.4
Outside of Adama town	192	59.6
**Maternal age at birth (in years)**		
15–19	12	3.7
20–24	80	24.8
25–2	141	43.8
30–34	64	19.9
35–39	21	6.5
40 years and above	4	1.2
**ANC Follow up**		
Yes	316	98.1
No	6	1.9
**Ultrasound scan during ANC**		
Yes	176	54.7
No	146	45.3
**Ultrasound scan during labour**		
Yes	313	97.2
No	9	2.8

### Obstetric characteristics

Regarding obstetrics characteristics, the mean parity was 2.6 (SD± 1.7) and the majority of mothers (98.1%) had 2–4 para. Approximately half of the mothers gave births at term and nearly the same number of mothers before term (less than 37 completed weeks). While half of the mothers gave birth through spontaneous vaginal birth, 46.6% of them gave birth by caesarean section. Moreover, the commonest indication for caesarean birth was found to be fetal mal-presentation (47.7%) followed by non-reassuring fetal heart rate patterns (NRFHRP) [10.5%] (Table 2).

**Table 2:** Obstetrics characteristics of the twin-births recorded at AHMC, Ethiopia.

Variables	Frequency	Percentage
**Parity**		
Para(I)	104	32.3
Para(II-IV)	183	56.8
Para(V & above)	35	10.9
**Gestational age at birth**		
Less than 37 weeks	150	46.6
37–40 weeks	154	47.8
Greater than 40 weeks	18	5.6
**Mode of birth**		
Spontaneous Vaginal delivery	160	49.7
Cesarean section	150	46.6
Assisted or complete breech extraction	2	0.6
Instrumental	1	0.3
Other combinations	9	2.8
**Indication for caesarean birth**		
Mal-presentation	73	47.7
Previous caesarean section scar	15	9.8
Arrest or protraction	13	8.5
NRFHRP	16	10.5
Cord prolapse	8	5.2
Others (APH, HDP, PROM)	28	18.3

**Note:** APH, Antepartum Hemorrhage; HDP: Hypertensive Disorders of Pregnancy, PROM: Premature Rupture of Membranes; NRFHRP: Non-reassuring Fetal Heart Rate Patterns.

### Adverse perinatal outcome

Of the total 322 paired (twin) birth records (644 individual birth records), 199 (61.8%) paired births had at least one adverse perinatal outcome on one or both twins. The commonest adverse perinatal outcomes observed in this study were low birth weight (50.8%) followed by preterm birth (46.6) and NICU admission (24.7%). As expected although, 24.5% of newborns had a low one-minute Apgar score, only 5.0% of them had a low five-minute Apgar score. Table 3 presents these adverse perinatal outcomes and others.

**Table 3:** Percentage distribution of adverse perinatal outcomes.

Adverse perinatal outcomes	Frequency	Percentage
Low birth weight	46.6	25
Preterm birth	46.3	23
Admission to NICU	24.7	12
Low Apgar score at 1^st^ minute	24.5	12
Mal presentation	21.4	11
Discordant twin	18.9	9
Still birth	4.7	2
Low Apgar score at 1^st^ minute	4	2
Early neonatal death	2.5	1
Congenital anomaly	0.6	0
Locked twin	0.3	0
Conjoined twin	0.3	0

### Factors associated with perinatal outcome

In a multivariable logistic regression model, factors such as younger maternal age and not having an ultrasound scan during ANC follow-up were significantly associated with Adverse perinatal outcomes after controlling for confounding factors. The odds of having adverse perinatal outcomes among twin births recorded to mothers within the age range of 15–24 years were four (AOR = 4.1, 95% CI; 1.3, 12.5) times higher than those births recorded to mothers within the age range of 35 years and above. In addition, not having ultrasound scan during ANC follow-up increased the odds of adverse perinatal outcomes by two folds (AOR = 2.0, 95% CI: 1.2, 3.1) (Table 4).

**Table 4:** Multivariable logistic regression analysis for factors associated with adverse perinatal outcomes.

Factor	Adverse perinatal outcomes		COR with 95% CI	AOR with 95% CI
	Yes	No		
	n (%)	n (%)		
**Place of residence**				
Adama town	71 (35.9)	59 (47.6)	Ref.	Ref.
Outside of Adama town	127 (64.1)	65 (52.4)	1.3 (0.8, 2.1)	
**Maternal age at birth**				
15–24	60 (18.6)	32 (9.9)	3.9 (1.3, 11.7)*	4.0 (1.3, 12.5)*
25–34	117 (36.3)	88 (27.3)	2.8 (0.9, 8.9)	3.2 (1.0, 10.9)
35 and above	21 (6.5)	4 (1.2)	Ref.	Ref.
**Parity**				
Primipara	67 (33.8)	35 (28.2)	Ref.	Ref.
Multiparas	131 (66.2)	89 (71.8)	1.1 (0.7, 1.9)	
**ANC follow up**				
Yes	195 (98.5)	120 (96.8)	2.3 (0.3, 19.8)	
No	3 (1.5)	4 (3.2)	Ref.	Ref.
**Ultrasound scan during ANC**				
Yes	97 (49.0)	79 (63.7)	Ref	Ref.
No	101 (51.0)	45 (36.3)	2.0 (1.2, 3.0)*	2.0(1.2, 3.1)*
**Ultrasound scan during labour**				
Yes	192 (97.0)	121 (97.6)	1	Ref.
No	6 (3.0)	3 (2.4)	1.420(0.778–2.592)	

## Discussion

The purpose of this study was to estimate the incidence of twin pregnancies and related-adverse perinatal outcomes and identify factors associated with adverse perinatal outcomes in twin pregnancies. The findings indicated that the incidence of twin pregnancies was 32.6 per 1000 births and close to 62% of twin birth records had at least one adverse perinatal outcome on one or both twins. Moreover, the findings revealed that younger maternal age (15–24 years) and not having ultrasound scan during antenatal care were associated with adverse perinatal outcomes.

The incidence of twin pregnancies observed in the current study (32.6 per 1000 births) was higher than the one reported in the studies conducted in Addis Ababa (24 per 1000) [19], Mekelle (14 per 1000) [20], and Gondar (14.9 per 1000) [21]. The high incidence of twin pregnancy in the current study could be partly attributed to the study setting. That is, since Adama Hospital Medical College is a referral hospital, it receives and manages mothers with a high-risk pregnancy from the local primary health facilities which probably might increase the number of twin pregnancies in the hospital. The findings of this study, however, in agreement with a findings reported from south west Ethiopia [22] and slightly lower than those reported in other African countries such as Niger, Congo, and Nigeria [8, 23].

In this study, the majority of twin births (44.4%) were recorded among mothers within the age range of 24–29 years. However, in a study conducted in southwest Ethiopia, it was found that more than half of twin births occurred among mothers who were under the age of 24 years [22]. Such variations in the sociodemographic characteristics related to twin pregnancies might be partly explained by the difference in socio-cultural issues such as early marriage.

Regarding the mode of births among twin pregnancies, it was found that 50% of mothers gave birth through spontaneous vaginal deliveries while 47% required caesarean section, which is comparable with the findings reported in the previous studies [24]. Twin pregnancy has been identified, alongside factors, such as advanced maternal age and maternal request without clinical indication, as one of the contributing factors for increasing rates of caesarean section [17].

In this study, it was found that the incidence of adverse perinatal outcomes was 61.8%, with low birth weight being the commonest adverse perinatal outcomes followed by preterm birth and NICU admission. The findings of these adverse perinatal outcomes, particularly low birth weight, were comparable with the one reported in a study conducted in Nigeria [25], but lower than the one reported in some Asian countries such as Thailand, India, and Korea [26, 27]. The observed discrepancies between the current findings and those reported from Asian countries might be due to contextual factors, such as the availability of advanced medical technologies that help for the survival of high-risk newborns (if born too early), increasing the rate of preterm, low birth weight, and NICU admission.

In this study, it was found that the odds of adverse perinatal outcomes were higher among the younger maternal age group. This finding is somewhat surprising and in contrast with findings from several studies [6]. However, similar findings were reported in a few studies [23, 28], warranting further study that explicitly addresses the effect of maternal age on adverse perinatal outcomes in twin pregnancies, preferably with a population-based sample. Not having an ultrasound scan during antenatal care also was found to be significantly associated with an increased risk of adverse perinatal outcomes in twin pregnancies. This might be due to the fact that pregnant women with twin pregnancies may not early identified and managed to avert adverse perinatal outcomes.

This study was not without some limitations. As it was based on retrospective hospital data, information on some sociodemographic and maternal-related variables that may influence the perinatal outcomes in twin pregnancies was not available, warranting a future study that addresses all the relevant variables in relation to the perinatal outcomes among twin pregnancies. Although antenatal ultrasound had been performed for most of the pregnant women, there were some limitations in the registry, meaning some variables including chorionicity were not clearly documented and were not assessed in this study. As the focus of this study was on fetus/newborn outcomes in twin pregnancies and some maternal-related variables were not available, the maternal outcomes in twin pregnancies were not included in this study, warranting further studies. Although assisted reproduction is less common in the study setting, we were not able to differentiate twins occurred after assisted reproduction, particularly in vitro fertilization (IVF). Furthermore, although the adverse perinatal outcomes in twin pregnancies were comprehensively assessed, the extent by which identified factors contribute for each adverse perinatal outcome might be slightly varies, indicating the need to address each perinatal outcome separately, particularly in settings where targeted-interventions are more feasible. Similarly, the adverse perinatal outcomes in twin pregnancies better compared with that of singleton pregnancies to indicate differential risks, whenever feasible.

## Conclusion

The incidence of adverse perinatal outcomes in twin pregnancies was high in this study. Factors such as younger maternal age and not having an ultrasound scan during antenatal care were found to be the strong predictors of adverse perinatal outcomes, indicating the for early screening and continuous monitoring of women with twin pregnancies to reduce the adverse perinatal outcomes.
